# Alarming implications: severe fever with thrombocytopenia syndrome and its biological vectors in the context of climate change

**DOI:** 10.3389/fmicb.2025.1544427

**Published:** 2025-07-25

**Authors:** Ze Chen, Manoj Baranwal, Albert A. Rizvanov, Mohammed Okely, Svetlana F. Khaiboullina

**Affiliations:** ^1^Hebei Key Laboratory of Animal Physiology, Biochemistry and Molecular Biology, Hebei Collaborative Innovation Center for Eco-Environment, Ministry of Education Key Laboratory of Molecular and Cellular Biology, College of Life Sciences, Hebei Normal University, Shijiazhuang, China; ^2^Department of Biotechnology, Thapar Institute of Engineering and Technology, Patiala, Punjab, India; ^3^Institute of Fundamental Medicine and Biology, Kazan Federal University, Kazan, Russia; ^4^Division of Medical and Biological Sciences, Tatarstan Academy of Sciences, Kazan, Russia; ^5^Entomology Department, Faculty of Science, Ain Shams University, Cairo, Egypt; ^6^Department of Microbiology and Immunology, University of Nevada, Reno, NV, United States

**Keywords:** severe fever with thrombocytopenia syndrome, climate change, biological vectors, *Haemaphysalis longicornis*, *Dabie bandavirus*

## Abstract

Severe fever with thrombocytopenia syndrome (SFTS) is an emerging tick-borne zoonotic disease. Since its identification in China in 2009, reports of SFTS cases have steadily increased, posing a significant threat to public health. This review summarizes the epidemiological characteristics of SFTS and its biological vectors, with a particular emphasis on the role of the tick vector *Haemaphysalis longicornis* in disease transmission. We also addressed the impact of climate change on the spread of SFTS and its biological vectors. With continued climate change, the spread of SFTS is likely to increase, consequently heightening the risk of infection. Furthermore, this review explores the prevention and control strategies for SFTS as well as future research directions, summarize the public health policies and the alleviation of the disease's impact on human health.

## 1 Introduction

In recent years, severe fever with thrombocytopenia syndrome (SFTS), caused by *Dabie bandavirus*, represents the most severe novel acute tick-borne disease. This disease primarily emerged in East and Southeast Asia, including China, South Korea, Japan, Vietnam, Thailand, and Myanmar (Cui et al., 2024; Chiho Kaneko, 2023; Kim and Lee, 2023; Rattanakomol et al., 2022, 2023; Lin et al., 2020; Kobayashi et al., 2020; Guo et al., 2016; Kim et al., 2013; Yu et al., 2011). The principal clinical feature of SFTS is fever, typically with body temperatures exceeding 38°C, accompanied by thrombocytopenia, which can lead to hemorrhages such as skin petechiae, ecchymosis, epistaxis, and gingival bleeding. Additionally, patients may have general malaise symptoms such as fatigue, nausea, vomiting, diarrhea and muscle aches. In the severe stage of the disease, multiple organ dysfunction could be present, including liver and kidney impairment as well as cardiac insufficiency, which can pose life-threatening risks. In 2017, the World Health Organization (WHO) listed it as one of the top ten infectious diseases requiring priority attention (Mehand et al., 2018).

*Dabie bandavirus* belongs to the order *Bunyavirales*, the family *Phenuiviridae*, and the genus *bandavirus*, which was formerly known as severe fever with thrombocytopenia syndrome virus (SFTSV; Hu et al., 2024). The virus is primarily transmitted by ticks, which become infected after biting host animals (wild and domestic animals) carrying the virus, and subsequently transmit it to humans. Additionally, human-to-human transmission may occur through contact with infected patients' blood or bodily fluids. In investigations, *Dabie bandavirus* and its antibodies have been detected in sheep, goats, cattle, and rodents, which could transmit the virus to humans (Wang J. N. et al., 2021). It was reported that two patients in Beijing, China, were infected with *Dabie bandavirus* through exposure to the blood of infected camels (Sun et al., 2024). Additionally, two workers at a Weihai farm were suspected of contracting the *Dabie bandavirus* while skinning infected animals (Li et al., 2024). Cases of direct transmission of the virus to humans by cats and dogs infected with the *Dabie bandavirus* have been reported in Japan (Kobayashi et al., 2020). In recent years, there have been numerous reports of clustering outbreaks of SFTS resulting from interpersonal transmission. More than 80 cases of interpersonal transmission of the *Dabie bandavirus* have been documented in South Korea and China (Hu et al., 2024).

Ticks, as biological vectors, have a significant influence on the transmission dynamics and epidemiological characteristics of *Dabie bandavirus* (Luo et al., 2015). The tick species *Haemaphysalis longicornis* was identified as the principal vector for the *Dabie bandavirus*, although other tick species or blood-sucking insects also contribute to its transmission. The incidence of SFTS is closely related to the habitat suitability of *H. longicornis* (Ding et al., 2023; Du et al., 2014; Zhang et al., 2022). For every 10% increase in its habitat suitability, the number of human SFTS cases will increase by 1.26 times (Ding et al., 2023). Additionally, in the context of climate change, *H. longicornis* populations are expected to exhibit one of the most significant responses among tick species, with measurable impacts on their geographical distribution, behavioral patterns, and population dynamics. Global warming is anticipated to drive northward expansion, and upward elevation shifts in their habitats, with models projecting a substantial increase in suitable areas by the 2080s (Ding et al., 2023). Furthermore, climate change is expected to alter both the seasonal distribution and spatial patterns of SFTS transmission (Ding et al., 2023; Du et al., 2014). Therefore, this review will focus on the epidemiological and biological characteristics of SFTS and its primary vector, *H. longicornis*, as well as emerging development trends. With great vigilance and attention, efforts should be made to effectively mitigate the ongoing spread of the disease and establish a solid line of defense to protect public health and ecological balance.

### 1.1 Epidemiology of SFTS

SFTS is a zoonotic disease that emerged in 2009 in Hubei and Henan provinces of Central China (Yu et al., 2011). *Dabie bandavirus* was first isolated from blood samples of patients and ticks in China in 2010. The disease has an incubation period of 7–14 days, followed by initial flu-like symptoms (Liu et al., 2014). Clinical symptoms of SFTS are fever, gastrointestinal symptoms, leukocytopenia and altered mental status (Liu et al., 2014; Seo et al., 2021). One of the most prominent laboratory findings are thrombocytopenia and leukopenia (Liu et al., 2014; Yu et al., 2011). In severe cases, patients could have acute kidney failure, encephalitis, respiratory failure and shock (He et al., 2021). There are four risk factors, such as older age, multiorgan dysfunction, elevated activated partial thromboplastin time and D-dimers, which were identified as contributing to patients' death (Fang et al., 2024). A similar conclusion was made in a study by Gai et al. (2012), in addition to four factors, elevated serum aspartate aminotransferase, lactate dehydrogenase, creatine kinase, and creatine kinase fraction, as well as the appearance of neurological symptoms were also named as contributing to patient's death. It is believed that the severity of the disease depends on the viral load, with fatal SFTS exhibiting the strongest correlation with high levels of viremia (Kim et al., 2024; Li et al., 2018). Also, the old age of the patient is a risk factor for severe and fatal SFTS (Liang et al., 2023; Zhao et al., 2022).

During the first outbreak in 2009, SFTS was diagnosed in two provinces, Hubei and Henan, in Central China (Yu et al., 2011). However, the disease spread rapidly, and it was diagnosed in 14 provinces in 2017 (Silvas and Aguilar, 2017). By 2018, SFTS was endemic in 25 provinces (Miao et al., 2021) with 7,721 total cases. Between 2010 and 2019, the majority of cases, 83.47%, were diagnosed in Lai Zhou, Penglai, Zhaoyuan, Haiyang, and Qixia (Hou et al., 2023). The number of cases increased after the outbreak in 2009. Between 2011 and 2016, 5,360 cases of SFTS were reported (Sun et al., 2017). In contrast, cases increased more than 3 times, to 18,902, from 2011 to 2021 (Liu et al., 2014).

Cases of SFTS are diagnosed between April and October (Chen et al., 2022; Cui et al., 2024). The highest incidence rate is documented in May, June, and July (Sun et al., 2017). There appears to be an early peak and longer duration of outbreak in the Southern provinces of China (Sun et al., 2017) compared to those in the North of China. Ticks could explain the seasonal pattern of SFTS, the primary vector transmitting the virus lifecycle. According to a study by Kang et al. (2022), the earliest time when *H. longicornis* ticks were captured was March, when they emerged from winter hibernation. This tick species could be captured until November when many tick larvae were found between September and October. These seasonal changes in the tick's lifecycle could explain the high incidence rate of SFTS between April and October.

SFTS is diagnosed in several countries such as China, South Korea, and Japan (Kim et al., 2013; Takahashi et al., 2014; Yu et al., 2011). By September 2018, 13,259 SFTS cases were reported in these countries (Miao et al., 2020). In 2017, the emergence of SFTS in Vietnam was reported (Tran et al., 2019). The spread of the disease is linked to the habitat of the *H. longicornis* ticks, native to Eastern Asia (Zhao et al., 2020). This tick species is also reported in Australia and New Zealand (Hoogstraal et al., 1968). In 2018, *H. longicornis* ticks were found on a sheep with no history of being outside the United States (Rainey et al., 2018). These data indicate a significant gap in our understanding of the longhorn tick habitat, which could extend beyond eastern Asia. This tick species could threaten livestock and humans around the globe.

In a study by Cui et al. (2024), an increased notification rate was documented in all endemic countries between 2009 and 2021; however, it was found significant only in China. It is believed that the Summer-Fall outdoor activities, such as farming and traveling, could contribute to outbreaks in those countries (Huang et al., 2021; Liu et al., 2023).

SFST clinical manifestation differ in China, South Korea and Japan. Variations in SFST symptoms and mortality rate could be explained by genotype of the circulating virus. There are six genotypes of *Dabie bandavirus* (A-F; Casel et al., 2021). Genotype B was found dominant in South Korea and Japan (Yun et al., 2020; Fu et al., 2016). In contrast, genotypes F, A, and D were shown dominant in mainland China (Fu et al., 2016). It should be noted that B genotype *Dabie bandavirus* identified in Zhoushan Islands, China, suggesting that virus origin could be Japan or South Korea (Fu et al., 2016). It was suggested that the *Dabie bandavirus* genetic variants could explain lower mortality rate in China compared to that in South Korea and Japan. The mortality rate associated with SFST in South Korea is 21.6%, while in Japan, the rate is 27% (Zhan et al., 2017; Yun et al., 2020). In contrast, the mortality rate from SFST in China is lower ranging from 5.3 to 16.2% (Hou et al., 2023; Huang et al., 2021).

Analysis of *Dabie bandavirus* genotypes in South Korea patients revealed high mortality rate among patients infected with B variant (44.4%) compared to that of A variant (10%; Yun et al., 2020). Similarly, B genotype was more frequently isolated from SFST patients compared to A genotype. Interestingly, a high mortality rate was found in patients infected with F genotype (44.4%), although the incidence rate was lower than that of B genotype. The higher severity and mortality of B genotype of *Dabie bandavirus* was confirmed using ferret model. The study demonstrated hospitalization rates of 50–51% and mortality rates ranging from 26.8 to 40%. Additionally, viral load is observed at 3.59–3.64 log copies/mkL. Levels of interferon α (IFNα), interleukin-10 (IL-10), interferon γ-induced protein 10 kDa (IP10) have been closely related to mortality (Kwon et al., 2024).

The B-2 genotype showed the highest incidence (48 of 133 cases) and a significantly higher mortality rate (21 of 48 patients, 43.8%) than the other genotypes. The F genotype also showed a high mortality rate (4 of 9 cases, 44.4%), although the incidence rate was lower than that of B-3 (8 of 28 cases) and B-1 (3 of 16 cases) genotypes. In addition, the A genotype showed the lowest mortality rate (1 of 10 cases) compared with the other genotypes (Yun et al., 2020). Also, The B-1 virus showed the most efficient replication compared with the other viruses in young ferrets and 100% mortality in old ferrets. In contrast, no mortality in young and 40% mortality in ferrets infected with A virus (Yun et al., 2020).

The genotypes F, A, and D were dominant in mainland China. Additionally, seven types of *Dabie bandavirus* genetic reassortants (abbreviated as AFA, CCD, DDF, DFD, DFF, FAF, and FFA for the L, M, and S segments) were identified from 10 strains in mainland China. Genotype B was dominant in Zhoushan Islands, Japan and South Korea, but not found in mainland China. Phylogeographic analysis also revealed South Korea possible be the origin area for genotype B and transmitted into Japan and Zhoushan islands (Fu et al., 2016).

Another mortality risk factor for SFST is linked to a patient's status. Older age, APACHE II score and use of vasopressive therapeutics are identified as independent factors for fatal SFST outcome (Yang et al., 2023). Also, significantly low platelet counts, high serum creatine kinase-MB, ALT and AST were commonly found in fatal SFST (Fei et al., 2023; Yang et al., 2023; Gai et al., 2012). It was indicated that the fatal SFST is characterized by deterioration of clinical and laboratory parameters in the late stage of the disease, when patients present with disturbed hemostasis, disseminated intravascular coagulation and multi-organ failure (Gai et al., 2012). The older age is a consistent risk factor for SFST mortality (Fang et al., 2024; Nie et al., 2020). It was suggested that the decline in the immune system function could contribute to fatality (Fang et al., 2024). Age being an important risk factor was also confirmed using ferret model, where old had substantially higher mortality rate compared to young mammals (Park S. J. et al., 2019). Similar to fatal SFST, old *Dabie bandavirus* infected ferrets had high serum levels of AST and ALT. Additionally, aged mammals presented with multiorgan virus dissemination.

*Dabie bandavirus* can contribute to the severity of the disease and fatal outcome. It was shown that nucleocapsid protein (NP) can suppress the interferon γ (IFN-γ) response (Wu et al., 2014; Ning et al., 2015). Additionally, the *Dabie bandavirus* could interfere with autophagy using it to facilitate virus replication (Yan et al., 2022). SFST infection can cause lymphopenia (Yang et al., 2023). Interestingly, the differential analysis revealed that the number of CD4+ lymphocytes was substantially lower in fatal SFST (Li et al., 2018). Among these leukocytes, the T helpers (Th) 1 and 2 as well as regulatory T cells were lower in deceased patient (Li et al., 2018). In contrast, Th17 lymphocyte counts were higher in fatal compared to non-fatal SFST.

Excessive activation of proinflammatory cytokines could contribute to pathogenesis of fatal SFST infection. Increased serum levels of several cytokines, including tumor necrosis factor (TNF-α), IFN-γ, IP-10, IL-10, IL-6, macrophage Inflammatory Protein-1 alpha (MIP-1α), IL-8, IL-15, granzyme B, heat shok protein 70 (HSP70), granulocyte colony stimulating factor (G-CSF), IL-1-RA, and monocyte chemoattractant protein-1 (MCP-1) in severe compared to mild forms of the disease were demonstrated in multiple studies (Song et al., 2017). The role of TNF-α and IL-6 is pathogenesis of fatal SFST is supported by finding that high viremia, low platelet counts and multiorgan failure correlate with high cytokine serum levels (Song et al., 2017). Elevated serum levels of TNF-α, IP-10, and IL-6 suggest a “cytokine storm”, condition characterized by over activation of pro-inflammatory cytokines which could damage tissues (Chen et al., 2021).

### 1.2 Biological vectors of SFTS

Biological vectors are the primary transmitters of the *Dabie bandavirus*. Vectors can affect the transmission process and epidemiological characteristics of SFTS (Luo et al., 2015). They significantly influence the transmission dynamics and epidemiological characteristics of SFTS (Luo et al., 2015). The tick species *H. longicornis* is identified as the principal vector for the *Dabie bandavirus*, although other tick species may also contribute to its transmission. In recent years, global climate change and human activities have expanded the habitat of *H. longicornis*, further increasing the risk of SFTS transmission (Ding et al., 2023). Its three-host life cycle and wide adaptability to hosts make controlling its population and transmission very difficult. Traditional control measures such as chemical pesticides require to be improved and often disrupt ecological balance. In this context, it is essential to strengthen biological and ecological research on vectors. This helps to understand the transmission mechanism of SFTS and provides a scientific basis for formulating effective control strategies.

#### 1.2.1 *Haemaphysalis longicornis*

##### 1.2.1.1 Ecological and biological characteristics of *Haemaphysalis longicornis*

*Haemaphysalis longicornis*, also called the Asian longhorned tick is a major vector and natural host for the *Dabie bandavirus*. This is an invasive tick species originating from East Asia, including China, Japan, South Korea, Vietnam and North Korea. In the mid-twentieth century, this species spread to other regions, such as Australia and Oceania (Hoogstraal et al., 1968). In 2017, the longhorned tick was first identified in the Americas, specifically in New Jersey, USA, and has rapidly dispersed to multiple states (Haddow, 2019; Pritt, 2020; Rainey et al., 2018; Wormser et al., 2020). This tick may have spread rapidly in the eastern United States through wild animals and livestock hosts (Stanley et al., 2020; Tufts et al., 2021). Individual parthenogenetic ticks have the capability to establish new populations, demonstrating a significantly higher reproductive efficiency compared to sexual reproduction. Evidence indicates that most long-distance invasions of longhorned ticks, such as those recorded in Australia, New Zealand, and the United States, are attributed to parthenogenetic populations (Egizi et al., 2020; Hoogstraal et al., 1968; Hyeon et al., 2021; Zhang et al., 2022).

Since 2007, this species has drawn the scientific community's attention as an essential vector for the emerging SFTS. The onset period of SFTS coincides with the peak activity period of *H. longicornis*. *Haemaphysalis longicornis* can thrive over a broad temperature range of −2 to +40°C and primarily inhabits secondary forests, mountainous areas, or the edges of hills. This tick species parasitizes a variety of hosts, including domestic animals such as cattle, horses, sheep, and goats, as well as wild animals such as deer, bears, badgers (*Meles meles*) and water deer (*Hydropotes inermis*). Moreover, this tick species can also infest humans. Larvae are mainly found on small wild animals such as Siberian chipmunks (*Eutamias sibiricus*) and birds such as the ring-necked pheasant (Chen and Yang, 2021).

This species undergoes four life stages: egg, larva, nymph, and adult. Except for the egg stage, it must feed on blood in each subsequent stage before molting into the next one or before laying eggs. *Haemaphysalis longicornis* exhibits both sexual reproduction and parthenogenesis. Parthenogenesis is a form of asexual reproduction that enables female ticks to establish new populations without fertilizing embryos, resulting in a more rapid population expansion than sexually reproducing ticks. Some data suggest a correlation between parthenogenetic populations and the transmission of *Dabie bandavirus* (Zhang et al., 2022).

It has a complex three-host life cycle. This tick can complete one generation in a typical year. The seasonal abundance studies on the bisexual population of *H. longicornis* in Hebei province, northern China, indicate that nymphs are primarily active from March to September, peaking in late April and early May. In contrast, adult ticks are most commonly found from April to September, peaking in late June to July. Females lay eggs predominantly in the warm summer months, leading to high rates of larval infestation between June and October, with peaks observed in mid-August to early September (Zheng et al., 2011). The oviposition period of female ticks is significantly shorter in May and June compared to July and August. The average daily rates of egg-laying during these months show a distinct difference, with lower daily oviposition rates observed in May and June, while the peak of oviposition in July occurs on the fourth day following oviposition (Zheng et al., 2011). Starving nymphs and adults survive the winter in nature.

Under standard laboratory conditions (23–28°C), the developmental cycle of *H. longicornis* is 83–144 days, and colder temperatures prolong its development period (Chen et al., 2024). Chen et al. (2012) synchronously studied several characteristics of parthenogenetic and bisexual populations of *H. longicornis*. The results suggested that most features of the two groups were similar, with only a few differences in characteristics. Parthenogenetic individuals have a wider genital apron, greater weight (excluding engorged females), and a longer development cycle of 134 days compared to bisexual individuals, which have a development cycle of 129 days. They also exhibit a lower hatch percentage and significant differences in nymphal premoulting, female feeding, preoviposition, and egg incubation periods.

Under laboratory conditions, the susceptibility of parthenogenetic and sexually reproducing female ticks to *Dabie bandavirus* is similar (Zhang et al., 2022). However, the distribution of parthenogenetic ticks strongly correlates with *Dabie bandavirus*, while bisexual ticks do not show the same correlation. The parthenogenetic ticks can reproduce rapidly and spread more widely compared to the bisexual ticks, allowing the SFTS virus to propagate quickly in East Asia. Overall, the unique reproductive biology of the Asian longhorned tick, particularly its parthenogenetic capability, is a crucial factor contributing to the rapid regional transmission of the *Dabie bandavirus*. A high proportion of parthenogenetic ticks were detected in migratory birds captured in *Dabie bandavirus* epidemic area, indicating that they may be transmitted through long-distance migration of these birds (Zhang et al., 2022). This assumption is supported by phylogeographic analysis of ticks collected from migratory birds (Zhang et al., 2022).

##### 1.2.1.2 The role of *H. longicornis* in the transmission of SFTS

*Dabie bandavirus* can be transmitted horizontally (from infected animals to ticks or from infected ticks to hosts) and vertically (from female ticks to their offspring; Zhuang et al., 2018). The virus can persist throughout the entire lifespan of the *H. longicornis*. Notably, this virus can be maintained in adult *H. longicornis* for up to 21 days, which is longer compared to persistence in other tick species (Zhuang et al., 2018). This extended retention of virus is crucial for the transmission of *Dabie bandavirus*.

Zhuang et al. (2018) demonstrated that after injection, *Dabie bandavirus* can be detected in salivary glands and ovaries of *H. longicornis* ticks for 12 days. These data indicate that *Dabie bandavirus* can infect and translocate within ticks. Moreover, this virus copies in the second generation of eggs were shown higher than that in the first generation of adult ticks prior to blood feeding. This could be explained by the blood feeding promoting the replication of *Dabie bandavirus* within the ticks. Viral RNA was detected in first- and second-generation ticks, indicating that *Dabie bandavirus* can be transmitted transovarially and transstadially. It was found in the tick's salivary glands and saliva, indicating that it could be transmitted through the host bites (Zhuang et al., 2018). These results significantly improve our understanding of the relationship between this tick species and *Dabie bandavirus* dissemination.

Additionally, the virus must evade the tick's innate immune response to persist and replicate in its vector. The antiviral innate immune response of ticks does not eliminate the virus. However, persistent viral infection does not have a significant impact on ticks when it comes to mitigating the negative effects of the infection (Talactac et al., 2021). *Haemaphysalis longicornis* can produce 22-nucleotide long virus-derived siRNAs (vsiRNAs) in response to infection by *Dabie bandavirus* (Xu et al., 2021). Xu et al. (2021) found that the tick Dicer2-like protein regulates the antiviral RNAi response, as knocking down this gene enhanced viral replication. Furthermore, authors demonstrated that viral suppressors of RNAi (VSR) proteins inhibit the production of vsiRNAs. These data indicate that ticks have an antiviral RNAi pathway that viruses managed to evade. This study suggests that the antiviral RNAi pathway of the long-horned tick is a target of virus evolutionary adaptations.

In conclusion, the transmission dynamics of *Dabie bandavirus* in *H. longicornis* include following steps: (1) During the bite, *H. longicornis* injects saliva while simultaneously absorbing blood through its mouthparts. Ticks can acquire the *Dabie bandavirus* by feeding on the blood of infected hosts or through transovarial and transstadial routes. (2) Initially, the virus persists within the tick's midgut, subsequently crossing the digestive epithelium to enter the hemocoel. (3) Later, it moves into the salivary glands by crossing the epithelium and invades the acini. (4) During the subsequent feeding period, *Dabie bandavirus* can be transmitted to new hosts via the saliva, which counteracts host hemostasis, inflammation and immune responses, thus facilitating pathogen infection of the host.

**Figure 1:**
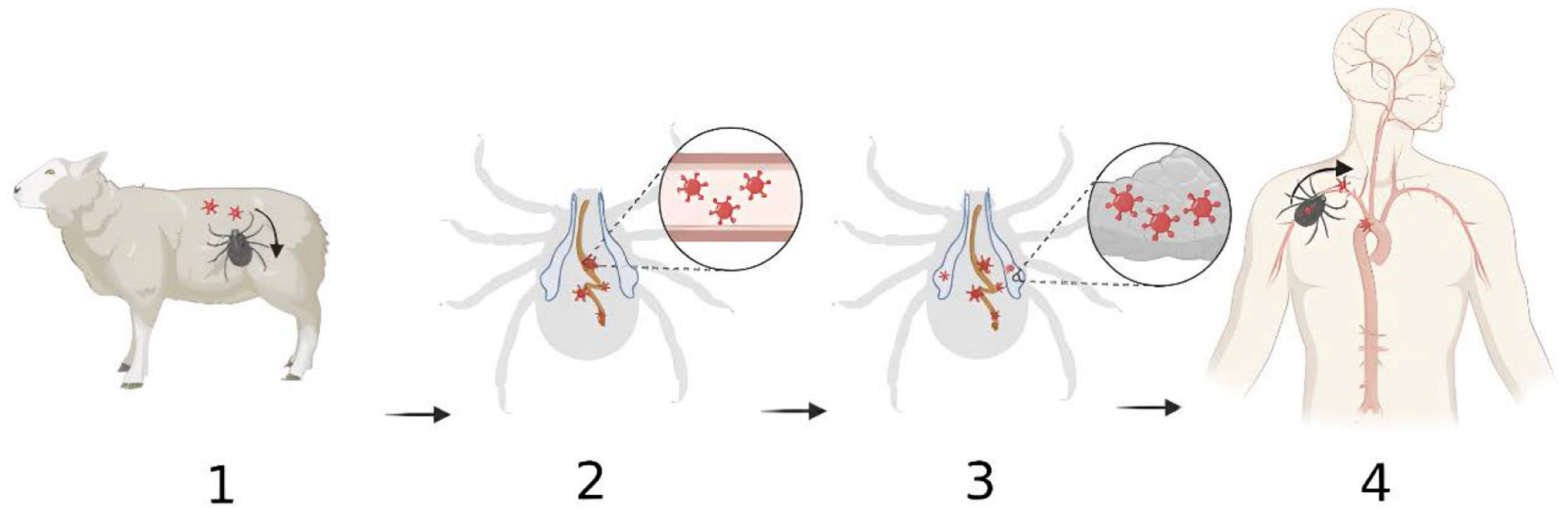
The transmission dynamics of *Dabie bandavirus* in *Haemaphysalis longicornis*. (1) Ticks become infected with *Dabie bandavirus* while taking blood meal from reservoir animals. Viruses enter the tick's gastro-intestinal tract; (2) The viruses initially replicate in the gastro-intestinal tract; (3) The viruses migrate from gastrointestinal tract to salivary glands where they continue to propagate; (4) The viruses are injected with saliva into the bloodstream during the feeding process. Created with BioRender.com.

Despite the important insights provided by laboratory research on the transmission dynamics of *Dabie bandavirus*, there are limitations to its findings in relation to real-world observations, primarily in several aspects: laboratory environments are typically subject to strict control, which does not reflect the complexity of natural environments; the animal models used in experiments, such as mice and guinea pigs, differ from the natural hosts of *Dabie bandavirus* (such as cattle and sheep) in physiological and immunological characteristics, which may affect the replication and transmission of the virus within the host; the viral dosage under experimental conditions is usually artificially controlled, while the viral load in actual environments is dynamically variable. Therefore, when interpreting laboratory research findings, it is essential to consider these limitations and analyze them in conjunction with actual data. Future studies should place greater emphasis on simulating natural environments and employ animal models that are closer to natural hosts to enhance the reliability and applicability of research outcomes (Figure 1).

#### 1.2.2 Other potential biological vectors

In addition to *H. longicornis, Dabie bandavirus* has been detected in various other biological vectors (Table 1). Through experimental transmission study by artificial infection, it was shown that the transmission capacity of *Dabie bandavirus* varies not only among different tick species but also across different periods and physiological states within the same species (Hu et al., 2020): (1) The duration of *Dabie bandavirus* carriage in *Ixodes sinensis* is 18 days, while it is 9 days for *Dermacentor silvarum* and 6 days for *I. persulcatus*. In contrast, the longest persistence of infection was 21 days in *H. longicornis*. (2) Five days after the injection of *Dabie bandavirus* in adult ticks, 100% of *I. sinensis* and *H. longicornis* ticks were found positive. In contrast, a significantly lower percentage of *I. persalcatus* ticks (66.7%) and *D. silvarum* ticks (33.3%) were positive for *Dabie bandavirus* RNA. (3) Although adult *D. silvarum* and *I. persulcatus* can carry *Dabie bandavirus*, they lack the capacity to transmit this virus via eggs and from nymphs to adults. Interestingly, *D. silvarum* capable of transstadial transmission of *Dabie bandavirus* in 81.25% cases, while *I. persulcatus* cannot. (4) The natural infection rate among free-living *H. longicornis* ticks is relatively low, while the infection rate of parasitic blood-sucking *H. longicornis* ticks is relatively high.

**Table 1:** Species detected with *Dabie bandavirus*.

**Tick species**	**Country**	**References**
*A. testudinarium*	ROK, Japan	Suh et al., 2016; Jo et al., 2019; Sato et al., 2021
*H. concinna*	China	Meng et al., 2015; Tian et al., 2017
*H. flava*	ROK, ROK, Japan	Yun et al., 2016; Jo et al., 2019; Sato et al., 2021
*H. formosensis*	Japan	Sato et al., 2021
*H. hystricis*	Japan	Sato et al., 2021
*H. megaspinosa*	Japan	Sato et al., 2021
*H. longicornis*	China, ROK	Liu et al., 2012; Jiang, 2012; Park et al., 2014; Xu et al., 2015; Luo et al., 2015; Meng et al., 2015; Wang et al., 2015, 2016; Liu et al., 2016; Xing et al., 2016; Yun et al., 2016; Jo et al., 2019; Yang et al., 2019; Shao et al., 2020; Zhang et al., 2022
*Ixodes nipponensis*	ROK	Ham et al., 2014; Yun et al., 2016; Suh et al., 2016; Jo et al., 2019
*I. sinensis*	China	Hu et al., 2020
*Rhipicephalus microplus*	China	Wang et al., 2015
*R. sanguineus*	China	Xu et al., 2015
Ticks unidentified	ROK	Yun et al., 2016; Lee et al., 2021; Seo et al., 2021
*Laelaps echidninus*	China	Wang et al., 2012
*Leptotrombidium scutellare*	China	Wang et al., 2012
Gadflies	China	Liu et al., 2012

*Dabie bandavirus* has also been detected in gamasid mites *Laelaps echidninus*, chigger mites *Leptotrombidium scutellare* and gadflies (Liu et al., 2012; Wang et al., 2012). However, viral RNA was not found in other blood sucking insects, such as mosquitoes, midges and sandflies (Liang et al., 2017; Tian et al., 2017; Yu et al., 2011). No viral RNA was detected in 5,900 mosquitoes collected from the SFTS patient's home (Yu et al., 2011). Also, it was shown that *Culex pipiens pallens, Aedes aegypti*, and *Anopheles sinensis* mosquitoes do not support virus replication (Liang et al., 2017).

### 1.3 The impact of climate changes on SFTS and its biological vectors

SFTS is a climate-sensitive infectious disease, as climate change has a profound impact on the biological vectors as well as the epidemic and transmission (Wang et al., 2024). Studies have confirmed that meteorological variables have nonlinear, delayed, and interactive effects on the incidence of SFTS (Zhang et al., 2019; Liu and Zhu, 2024; Wang et al., 2024; Zhan et al., 2017).

Liu et al. (2015) conducted a comprehensive analysis of the epidemiological characteristics, geographical variations, spatiotemporal clustering, and risk factors associated with SFTS in China from 2010 to 2013. Utilizing spatial scan statistics and the Boosted Regression Tree (BRT) model, they identified climate factors such temperature, rainfall, relative humidity, and sunshine hours as significant risk factors. Subsequently, Sun et al. (2018) employed various predictive models, including ARIMA, NBM, and GAM, to forecast the incidence of SFTS and determined a notable influence of temperature and relative humidity on the disease's occurrence. Among these models, the NBM demonstrated the most effective predictive performance. Their findings indicated that a unit increase in last month's SFTS cases leads to a 1.17% rise in occurrence, a unit increase in maximum temperature correlates with a 25.68% increase, and a unit increase in mean relative humidity results in a 10.31% increase in SFTS cases (Sun et al., 2018). Ding et al. (2023) used BRT and GAM models to forecast an increase in SFTS cases in mainland China from 2030 to 2089 compared to the 2010s, with regional variations. Specifically, the provinces of Liaoning and Shandong are expected to increase, whereas a decline may occur in Henan. Furthermore, potential outbreak zones are anticipated to emerge in Xinjiang and Yunnan (Ding et al., 2023). Additionally, Miao et al. (2020) utilized the ecological niche model alongside the BRT model to evaluate the global distribution of the tick species *H. longicornis* and identify potential hotspots for SFTS. Their analysis revealed that, beyond the established endemic areas, this tick species exhibits high suitability in regions such as the northeastern United States, New Zealand, parts of Australia, and several Pacific islands. Consequently, high-risk SFTS areas are primarily concentrated in east-central China, most of the Korean Peninsula, southern Japan, and northern New Zealand (Miao et al., 2020).

The dependance of SFTS incidence rate on meteorological factors could provide crucial information for resource allocation, planning the disease control in the context of climate change. The most critical factors will be discussed in this section.

#### 1.3.1 Temperatures

Among many factors, temperature is a crucial environmental factor influencing the incidence rate of ticks and SFTS. Both low and high temperatures inhibit tick host-seeking activities, affecting their habitat and duration of activity. When environmental temperatures decrease significantly, ticks respond behaviorally by withdrawing to the litter area to avoid freezing (Gray, 2008), entering states of quiescence and diapause (Gray et al., 2016) or synthesizing cryoprotectants, which may enhance their ability to tolerate cold and improve their survival during the winter (Neelakanta et al., 2010). Therefore, lower temperatures extend the developmental cycle duration, consequently increasing tick mortality rates (Estrada-Peña et al., 2012). However, rapid cold hardening (RCH), a type of phenotypic plasticity that enables ectotherms to quickly enhance their cold tolerance in response to brief chilling (lasting minutes to hours), improves cold survival but shortens lifespan due to increased metabolic costs (Teets et al., 2020; Wang et al., 2017b). This relationship explains why warmer climate conditions generally favor the establishment of permanent tick populations (Estrada-Peña et al., 2012).

However, the relationship between temperature and tick populations is not linear (Estrada-Peña et al., 2012). While moderate temperature increases may initially benefit ticks by accelerating development and extending their active periods, extreme heat can significantly hinder survival and reproductive success through accelerated water loss, especially for certain life stages. Laboratory research of *Ixodes scapularis* indicates that temperatures surpassing 32°C can adversely affect tick survival, hindering the molting process of larvae and nymphs into the next life stages (Eisen and Eisen, 2024). The mortality effect of high temperatures is particularly pronounced when combined with low humidity conditions, resulting in a dual stress environment that further reduces survival potential (Eisen and Eisen, 2024). The relationship between thermal stress and survival is particularly important when considering climate change projections, as expanding periods of elevated temperatures could substantially impact tick population dynamics across various regions (Bouchard et al., 2019).

Studies have demonstrated that there is also a complex nonlinear relationship between temperature and SFTS (Wang et al., 2024; Zhang et al., 2019; Wang et al., 2017a). Feng et al. (2017) isolated *Dabie bandavirus* strain ZJ2013-06 from a patient with SFTS who did not exhibit fever symptoms in Zhejiang, China. This strain exhibited significant genetic divergences from the predominant strains circulating in China. Temperature sensitivity tests found that compared to 37°C, ZJ2013-06 had limited replication at 39°C, with a significant decrease in viral load (about 100-fold). Through adaptive culture at 39°C, they successfully obtained the ZJ2013-06-P7 strain, which exhibited a reverse mutation at the 1616th amino acid residue (transitioning from aspartic acid to serine). This mutation enabled ZJ2013-06-P7 to lose the temperature sensitivity characteristic of the original strain, restoring its replication capability to levels similar to those of the typical strain NB24/CHN/2013. Consequently, the reverse mutation is the key to the temperature sensitivity of the ZJ2013-06 strain (Feng et al., 2017). Analysis by Du et al. (2014) indicated that the annual mean temperature favorable for the outbreak of SFTS ranges from 11.6 to 12.8°C. The majority of SFTS cases are reported between May and October. This period coincides with optimal temperature conditions for tick activity, thereby indirectly indicating the role of temperature in promoting *Dabie bandavirus* transmission. In regions characterized by elevated temperatures and humidity, ticks are prevalent for extended periods, and cases of SFTS documented longer. For instance, in Jiangsu Province, SFTS cases typically emerge between March and April, reach their peak from May to August, and subsequently decline and ultimately vanish by November (Zhang et al., 2019), suggesting that warmer months are more favorable to spread of *H. longicornis* and the transmission of the disease. Studies have shown that a temperature range of 18–35°C may increase tick population density and facilitate further reproduction of their offspring (Heath, 2016).

The maximum temperature of the warmest month is also a key environmental factor, with a suitable range between 32.8 and 34.2°C (Zhang et al., 2019). The exposure-response curve with an inverted “U” shape indicates that the risk of SFTS natural foci presence increases initially followed by decline as temperature changes.

In summary, temperature is a crucial factor influencing *Dabie bandavirus* infection and pathogenesis. The sensitivity of *Dabie bandavirus* to temperature may be related to its pathogenic mechanism and clinical manifestations. Elevated temperatures within an appropriate range enhance the replication and transmission efficiency of this virus within its vector (Zhang et al., 2019). Elevated temperatures also contribute to a higher survival and reproductive success rate of *H. longicornis* and accelerate metabolism leading to a shorter life cycle and a growth in population size, which further influences the transmission dynamics of *Dabie bandavirus*. Moreover, the temperatures that favor tick growth also promote increased human activities, resulting in a higher likelihood of human-tick interactions and, consequently, an elevated incidence of SFTS.

#### 1.3.2 Precipitation

Precipitation is another crucial climatic factor that significantly influences SFTS and *H. longicornis*. Moderate levels of precipitation create an environment favorable for ticks' survival and facilitate the *Dabie bandavirus* transmission. In contrast, excessive or insufficient precipitation may negatively affect tick activity and reproduction. An optimal range of precipitation (100–400 mm), combined with abundant vegetation, creates ideal living conditions for *H. longicornis*. The amount of precipitation during the dry season serves as a significant driving factor for the transmission of *Dabie bandavirus* (Liu et al., 2014). The influence of precipitation on SFTS and ticks can be understood through the following several interconnected aspects.

First, precipitation directly affects the habitat suitability and survival strategies of ticks, which in turn influences the spread of SFTS. The influencing mechanism varies across different regions (Ding et al., 2023; Duan et al., 2023; Mo et al., 2025; Wu et al., 2020). Favorable humidity conditions enhance the survival and reproduction rates of ticks, thereby facilitating the establishment of their populations (Berger et al., 2014; Tsunoda, 2008; Yoder et al., 2008). For instance, *I. ricinus* requires >92% relative humidity (RH) for off-host survival, while desert-adapted *Hyalomma asiaticum* tolerates 80% RH (Lees, 1946; Balashov, 1960). Precipitation sustains these thresholds through microhabitat exploitation, such as *I. ricinus* alternates between dry vegetation and moist ground litter to rehydrate (Lees and Milne, 1951), whereas *Hy. asiaticum* retreats to rodent burrows (84–100% RH) during droughts (Balashov, 1960). Ticks also absorb atmospheric water vapor above their critical equilibrium humidity (CEH: 75–94% RH), a process accelerated by precipitation-driven humidity (Rudolph and Knülle, 1974, 1978). These adaptations—coupled with physiological mechanisms like cuticular waterproofing and water vapor absorption via mouthparts—enable ticks to balance hydration across life stages, from parasitic phases (excreting excess blood meal water via salivary glands or coxal organs) to prolonged fasting periods (Gassner et al., 2011; Medlock et al., 2013).

Second, precipitation directly affects the ticks' biogeographic constraints. Behavioral plasticity and evolutionary adaptations to humidity gradients ultimately define tick biogeography. Species like *I. ricinus* exhibit vertical migration between vegetation and humid litter layers to mitigate moisture loss during host-seeking (Medlock et al., 2013), while desert species avoid daytime aridity by sheltering in burrows (Balashov, 1960). Crucially, distribution limits are dictated not by CEH thresholds but by cuticular permeability—a trait determining interspecies tolerance to environmental humidity. These patterns underscore how precipitation-driven humidity interacts with species-specific physiological and behavioral traits to shape global tick distributions (Milne, 1943).

Third, precipitation indirectly affects the habitats of small animals, such as rodents, which are essential hosts for ticks (Agulova et al., 2016; Gubler et al., 2001). Fluctuations in rainfall correlate closely with the population dynamics of these small mammals (Barros et al., 2018; Dhawan et al., 2018). Increased precipitation can lead to higher breeding rates among hosts, offering more opportunities for ticks to infest, which can result in an elevated risk of SFTS transmission (Agulova et al., 2016; Wang et al., 2024). Moreover, the incidence rate of SFTS demonstrates distinct seasonal variations, typically peaking during periods with increased precipitation and elevated temperatures (Wu et al., 2020). Data suggests that higher rainfall creates optimal conditions for tick survival and may also enhance the SFTS transmission potentially through an increase in host animal populations.

Lastly, it is important to note that the effects of precipitation are related to the occurrence of SFTS. Numerous studies demonstrated that humidity significantly influences on SFTS (Duan et al., 2023; Mo et al., 2025; Sun et al., 2018; Wang et al., 2024). Conversely, Wu et al. (2020) found that the occurrence of SFTS was relatively insensitive to precipitation, suggesting that the influence of precipitation may be affected by geographical location and time period. Furthermore, the relationships between precipitation and infection rate of SFTS are non-linear and SFTS may exhibit delayed consequences (Wang et al., 2024). This suggests that the impact of rainfall may not be immediately reflected in the number of SFTS cases. Humidity and precipitation affect the survival activities of ticks and the spread of SFTS under extreme conditions, and the specific influencing mechanism varies from region to region (Deng et al., 2022).

In conclusion, humidity plays a crucial role in the occurrence of SFTS, with a generally positive correlation but also showing non-linear relationships and varies regionally. It indirectly influences the transmission of SFTS by affecting ticks. Additionally, humidity is also related to the incidence rate of SFTS cases, but this relationship is influenced by a variety of other factors, including temperature, geographical location, and seasonal variations. Understanding these relationships is crucial for predicting and preventing the spread of SFTS.

#### 1.3.3 Climate-induced ecological landscape alterations

Climate-driven ecological changes are expected to significantly alter the natural hosts and transmission pathways of the *Dabie bandavirus*. Notably, climate-induced alterations in ecological landscapes are likely to impact the geographical distribution of *H. longicornis*. Furthermore, the effects of climate change on habitats will influence tick populations, host animals, and the overall transmission dynamics of SFTS (Okely et al., 2025).

Collected data highlights key elements regarding how climate factors affect SFTS transmission dynamics. First, climate conditions and tick density significantly influence the transmission ecology of SFTS (Deng et al., 2022). Shifts in temperature and precipitation patterns can result in considerable habitat alterations (Weiskopf et al., 2020). Warmer temperatures and increased rainfall create more favorable environments for tick proliferation, expanding tick habitats into previously inhospitable regions, which subsequently increases their geographical range and capacity to transmit pathogens (Dantas-Torres, 2015). Additionally, rising temperatures accelerate the life cycle of ticks, lengthening their active seasons and consequently elevating tick populations. Resulted an increased tick population heightens the likelihood of encounters between ticks and their host animals, amplifying the risks of disease transmission (Chen et al., 2024).

Second, tick-host dynamics are critically intertwined with climatic changes. Ticks are parasites of various hosts, which can increase the chances of *Dabie bandavirus* transmission to humans. The distribution patterns of these host species may be altered by climate change, impacting SFTS transmission rates (Iijima et al., 2025).

Geographical factors, such as altitude, also influence *Dabie bandavirus* transmission by affecting the living conditions of ticks and the virus's transmission dynamics within these vectors (Liu et al., 2015; Sun et al., 2021). The interplay among ticks, hosts, and environmental changes creates complex transmission pathways for diseases such as SFTS. Expanding tick and host populations can elevate the frequency of transmission events, increasing the risk of SFTS in previously unaffected regions (Wang J. N. et al., 2021). Habitat disturbances resulting from climate fluctuations may alter host behavior and distribution, further complicating transmission dynamics (Wang J. N. et al., 2021). In summary, climate change can profoundly affect SFTS transmission pathways and associated risks by modifying the distribution of ticks, host animals, and their interactions. Further investigation into these intricate ecological processes is vital for predicting and responding to the repercussions of climate change on SFTS transmission.

Lastly, environmental changes driven by climate are affecting the seasonal patterns of tick activity and SFTS outbreaks. Longer and warmer seasons lead to higher survival rates for both ticks and their hosts, prolonging transmission seasons (Jang et al., 2024). Such shifts may result in increased incidents of SFTS during atypical months that extend beyond traditional peak seasons, posing challenges for public health monitoring and response efforts. Adaptation and dispersal of *H. longicornis* to changing ecological landscapes was reported correlating with the increased spread of SFTS in Southeast Asia (Pérez et al., 2024). Furthermore, the genetic and ecological diversity of *H. longicornis* plays a crucial role in its capacity to transmit SFTS. Recent studies demonstrated that ecological landscape factors, with significant public health implications, influence this virome diversity (Ye et al., 2024). Variability in environmental conditions affects the interactions between viruses and their vectors, underscoring the importance of landscape genetics in understanding transmission dynamics (Ye et al., 2024; Kim et al., 2022).

Environmental factors such as temperature, precipitation, and habitat type can establish ecological niches favorable to tick survival and proliferation, increasing the risk of SFTS transmission (Pérez et al., 2024). Additionally, understanding the overwintering ecology of *H. longicornis* is critical for managing SFTS risks. Jung and Lee (2023) found that the unique characteristics of overwintering habitats influence tick survival and the potential for virus transmission in subsequent seasons. It appears that climate change will continue to reshape habitats suitable for *H. longicornis*. Various climate scenario forecasts a shift in the geographical distribution of these ticks, potentially expansion into new regions (Pérez et al., 2024). Overall, the interplay between ecological landscape changes and the biology of *H. longicornis* is essential for understanding and mitigating SFTS impacts. Further research into these complex interactions and the anticipated effects of climate change will be crucial for public health preparedness and response strategies.

### 1.4 Prevention and control strategies in the context of climate change

Mitigating the potential impacts of climate change on SFTS and its vectors necessitates coordinated efforts from various stakeholders.

#### 1.4.1 Public health departments

Public health agencies are crucial in monitoring and responding to emerging infectious disease threats, such as SFTS, exacerbated by climate change.

Asian longhorned ticks feed on multiple hosts, such as livestock, white-tailed deer, sheep, goats, hares, and dogs (Maestas et al., 2020). Also, ticks were found on birds (Pandey et al., 2022). This wide range of hosts makes *H. longicornis* highly adaptive to a changed environment and extremely mobile. Management of this tick species presents a challenge as these ticks can propagate using parthenogenesis (Wang T. et al., 2021). Also, they have a complex lifecycle which includes several stages with different susceptibility to control measures. The current tick control approaches aim to map the tick's spread and use acaricides and personal protection. The tick map is a tracking system led by Agricultural Research Service (ARS) to identify the area where ticks are endemic (Baldwin et al., 2022). This map allows ARS to investigate the tickborne disease outbreaks and predict the location of the next one. It can also help identify farms at risk before ticks get there. This map is an excellent tool for the United States Department of Agriculture (USDA), a leading office to monitor and control tickborne diseases. This effort involves the Partnerships for Data Innovations (PDI) team, which connects technology leaders, researchers, and USDA to address the challenges in the agricultural community. The result of this effort, a tick map, becomes a powerful new tool that provides real-time awareness of the efforts to fight the tick.

Surveillance is a powerful tool for analyzing ticks' distribution in different habitats and the presence of various stages of the arthropod lifecycle. Two commonly used methods are “dragging” and “flagging”. For “dragging”, a white cloth is dragged behind a researcher walking through a habitat, while a white cloth is waved on the ground when “flagging”. When ticks are identified near the farm, livestock or human settlements, a chemical control could be used to manage the arthropods. Several pesticides were found to be effective in a study by Park G. et al. (2019). Studies also demonstrated pyrethroids' efficacy in controlling ticks' nymphal stage (Lee et al., 2015; Park G. et al., 2019). Also, continued use of acaricides was suggested to target nymphal and adult stages, which could reduce the number of females laying eggs. Several acaricides could be used as dips and sprays for animals (Mutavi et al., 2021; Oda et al., 2019; Watts et al., 2016).

Tick bite prevention reduces human exposure, which is essential to control the spread of infectious diseases. The Centers for Disease Control and Prevention recommendations include using Environmental Protection Agency (EPA)-registered insect repellents (Centers for Disease Control and Prevention, 2024). The efficacy of CDC-suggested repellents against nymph was confirmed in a study by Foster et al. (2020). Wearing light-colored cloth preventing tick attachment is also recommended to prevent tick bites (Stjernberg and Berglund, 2005; US Environmental Protection Agency, 2024). Additionally, CDC recommendations state that showering and body inspection are required every time after having an outdoor activity (Centers for Disease Control and Prevention, 2024).

#### 1.4.2 Environmental and climate scientists

Global warming increases the risk of tick bites for humans and animals. This could result from the spreading of animals into new habitats and increased human activities such as travel, tourism and trade. The rising temperature is the main factor contributing to the increased risk of tick exposure (Ogden and Lindsay, 2016). Higher temperatures support the growth of the tick population (Gasmi et al., 2018). The warmer temperature was shown to expedite oviposition, egg development and interstadial development rate in ticks (Li et al., 2016; MacLeod, 1935; Randolph et al., 2002). Increased temperature also prolongs the seasonal activity of ticks (Gilbert et al., 2014). Climate changes also promote the growth of the tick population by providing an abundance of additional hosts in new habitats.

One of the most pressing challenges for environmental researchers is to predict changes in the suitable area for tick propagation. In a study by Namgyal et al. (2020), the East Coast and West Coast states were found suitable for the *H. longicornis* habitat. These findings are supported by data collected by using maximum entropy distribution modeling (Raghavan et al., 2019; Rochlin, 2019). *Haemaphysalis longicornis* appears highly adaptive and can survive in various climates. They can sustain a wide range of temperatures (Heath, 2016; Zhao et al., 2020). However, low humidity could limit ticks' survival as larva and adult stages require moisture (Knülle and Rudolph, 1982; Zhao et al., 2020). Another factor contributing to *H. longicornis* spread is the presence of tall grass and meadows (Schappach et al., 2020).

Tick control measures were tested in multiple studies. For example, a randomized, replicated, fully crossed, placebo-controlled, masked test was conducted by Keesing et al. (2022) to evaluate the efficacy of two environmentally safe interventions. The Tick Control System (TCS) and Met52 fungal spray were used separately or combined to measure the reduction of risk for and incidence of tick-borne diseases in humans and pets in 24 residential neighborhoods. Authors stated that these approaches were effective only to reduce the risk of tick-borne disease in pets, while having limited effect on risk of human exposure. Similarly, limited effect of bifenthrin on risk of tik-borne disease in human was reported by Hinckley et al. (2016) in 2-year, randomized, double-blinded, placebo-controlled trial conducted in 3 northeastern states. It was suggested that these acaricides could have limited effect on ticks exposure because of human awareness of that risk and taking precautions.

Study using ecotonal woodchip borders reduce the density of host-seeking ticks along recreational trails in Ottawa, Canada was reported by McKay et al. (2020). It was reported that this ecologically friendly method reduced the abundance of questing ticks reducing the risk of human exposure. The efficacy of acaricide Ecotix was tested by Hezron et al. (2012) in Tanzanian short horn Zebu, Tanzania. Authors reported the efficacy of the treatment as well as economic benefits for farmers.

In 2007, the Center for Disease and Prevention established a TickNet, a public health network which includes academic institutions and state health departments to coordinate the surveillance, research, education and prevention of tick-borne diseases (Mead et al., 2015). This initiative partners with emerging infections programs from Massachusetts, Minnesota, New York and Connecticut to conduct a survey or commercial, clinical and state laboratories to evaluate the practices used to test for tick-borne diseases (Mead et al., 2015). Also, these partners quantify current costs for diagnosis and treatment of Lyme disease, a most common tick-born disease.

Tick surveillance program was established and funded by CDC in 2018 (https://www.cdc.gov/ticks/data-research/facts-stats/?CDC_AAref_Val=https://www.cdc.gov/ticks/surveillance/index.html). At that time states, counties and tribes used different approaches for tick surveillance, which affected the coordination of tick control measures. To improve the tick control practices CDC implemented a cooperative agreement to support five regional Center of Excellence in Vector-Borne Disease, in 2017 (https://www.cdc.gov/vector-borne-diseases/what-cdc-is-doing/centers-of-excellence-in-vector-borne-diseases.html?CDC_AAref_Val=https://www.cdc.gov/ncezid/dvbd/about/prepare-nation/coe.html). To continue this effort, CDC awarded four universities to serve as Centers of Excellence: University of Massachusetts Amherst, University of California-Davis, University of Florida, and University of Wisconsin-Madison. These Centers are conducting research to prevent tick bites and suppress the acari population. Also, they train public health entomologists to become experts in vector-borne diseases. Additionally, they become a center for gathering information on tick-borne diseases and acari control.

The Federal Framework on Lyme Disease Act, which was approved on December 16, 2014, is aimed to develop a Federal Framework on Lyme disease, which is an emerging tick-borne infection in Canada (https://www.canada.ca/en/public-health/services/diseases/lyme-disease/surveillance-lyme-disease.html). This framework is based on three principles: (1) surveillance, (2) education and awareness, and (3) guidelines and best practices. The initiative identifies a tick surveillance in public places as one of the essential elements in prevention of Lyme disease. The integrated surveillance data will provide timely updates on the changing distribution of tick vectors. Also, Regulatory Proposal PRO2018-01 was developed by the Government of Canada to update recommendations in the Pest Control product Act (https://laws-lois.justice.gc.ca/eng/acts/P-9.01/) on use of products applied on skin of pets to control ticks (https://www.canada.ca/en/health-canada/services/consumer-product-safety/pesticides-pest-management/public/consultations/regulatory-proposals/2018/pesticide-products-used-companion-animals/document.html). The Government of Republic of China released “Regulations on the Management of Pesticides” (State Council Decree 677). These Regulations concern the registration, production, distribution, and use of pesticides to control ticks (https://apps.fas.usda.gov/newgainapi/api/report/downloadreportbyfilename?filename=China%20Released%20the%20Regulations%20on%20the%20Management%20of%20Pesticides_Beijing_China%20-%20Peoples%20Republic%20of_4-19-2017.pdf). Also, Chinese Center for Disease Control and Prevention published guidelines to educate population on steps to reduce tick's exposure (https://en.chinacdc.cn/in_focus/202204/t20220412_258378.html). European Center for Disease Control and Prevention (ECDC) published personal protection measures against tick bites (https://www.ecdc.europa.eu/en/disease-vectors/prevention-and-control/protective-measures-ticks). Also, detailed information generated in collaboration with the VectorNet Entomological Network on biocide resistance in wild vector population is summarized on the ECDC site (https://www.ecdc.europa.eu/en/publications-data/biocide-resistance-wild-vector-populations-eu).

#### 1.4.3 Decision-makers and legislators

Decision-makers and legislators play a pivotal role in addressing the climate-SFTS-vector relationship.

Control of *H. longicornis* distribution is an important healthcare issue because they can be vectors for zoonotic diseases (Luo et al., 2015; Zhao et al., 2020). One of the most severe zoonotic viral infections transmitted by this tick species is SFTS. This disease can have a mortality rate of up to 35% (Yokomizo et al., 2022). STPS is endemic in Asia. However, the spread of tick vectors to North America makes SFTS a potential threat on the continent. This potential for an outbreak of SFTS in new areas requires attention from decision-makers. *Haemaphysalis longicornis* can also be a vector for livestock diseases. This tick species can transmit *T. orientalis* Ikeda, causing bovine theileriosis in Asia and North America (Butler and Fryxell, 2023; Egizi et al., 2020). *Haemaphysalis longicornis* is reported in the northeast, mid-Atlantic, and southeast of the US (Beard et al., 2018). This tick spreads rapidly due to the diverse host range and high reproduction rate (Ronai et al., 2020; Tufts et al., 2021). Currently, *H. longicornis* is not reported in Texas as a major livestock producer; however, the rapid spread of this species requires serious constant attention. The USDA Animal and Plant Health Inspection Service developed a “Complex Program for Monitoring *Haemaphysalis longicornis*, the Asian Longhorned Tick, Populations in the United States” (USDA, 2024). Monitoring is based on analysis of the status of occurrence. This could be done by tick identification using reference laboratories such as USDA National Veterinary Services Laboratories (NVSL). Also, state laboratories specializing in tick identifications can do additional identifications. Surveillance and distribution of ticks could be completed by active and passive techniques with GPS locations recorded. Collected data could be used for the analysis of outbreaks by state agriculture and public health agencies. Also, these data could be used to assess the risk and potential impact of *H. longicornis* on disease introduction and spread in the United States.

Initial detection of *H. longicornis* by state laboratories should be confirmed at the NVSL. During the investigation, a local USDA Veterinary Medical Officer (VMO) should provide a copy of the pest alert fact sheet to the concerned citizen, landowner, or producer. Measures of tick control should be identified and provided to local authorities. These measures include: (1) modification of landscape, (2) use of acaricides, and (3) repellents as well as biosecurity. Habitat modification is part of the integrated pest management plan for tick control. Since *H. longicornis* habitat in high grass and meadow areas (Schappach et al., 2020). Therefore, trimming grasses, removing trees, and placing mulch and surface barriers could reduce the survival of ticks. Additionally, temporal removal of the livestock could reduce the survival of all states of tick development. Several tick control acaricides are approved by the Environmental Protection Agency (EPA) and the Food and Drug Administration (FDA). Permethrins, phosmet, diflubenzuron, and lambda-cyhalothrin effectively killed exposed *H. longicornis* (Butler et al., 2021). The biosecurity includes personal protective equipment (PPE) to minimize exposure to ticks. These measures focus on protecting property from entry and spread of pests.

Biological control is an essential component of the integrated pest management (IPM) program (Samish et al., 2004). This approach utilizes pathogenic microbes to control ticks' population. Among these pathogens, fungi species were used to test acaricidal efficacy *in vitro* and *in vivo* (Alonso-Díaz and Fernández-Salas, 2021; Wadaan et al., 2023; Sullivan et al., 2022). The *Metarhizium anisopliae* and *Beauveria bassiana* species are most commonly used for pests control (Rajula et al., 2020; Bukhari et al., 2011). Biocontrol has multiple advantages compared to other types of acaricides. This form of control is targeted with limited toxic effect to the environment and other species (Graf et al., 2004; García-García et al., 2000). Microbes could remain in the environment providing sustainable tick's control (Estrada-Peña et al., 1990; Mesquita et al., 2023). Also, pathogenic microbes could target nymph and larvae stages of tick life cycle, which explains the high efficacy of tick's control (Wassermann et al., 2016; Alonso-Díaz and Fernández-Salas, 2021; Bonnet et al., 2021). Biocontrol approach could be an additional tool used by heath care departments to control tick's population and prevent the spread of infections.

A novel approach for ticks control based on gene editing was proposed (Nuss et al., 2021). Genetic manipulation could be used to affect the male-to-female ratio which was demonstrated in experiments using mosquito (Galizi et al., 2016). Similar approach could be used to control tick's population.

Public education is crucial for the surveillance effort. Increased public awareness of tick habitats and the diseases they transmit could reduce the risk of outbreaks. This information could be provided to veterinarians and pet owners through press releases and broadcasts. Also, information could be provided to the public on information sheets, distribution maps, etc.

## 2 Conclusions

Tick spread due to climate change is a significant challenge. The combined effects of epidemiologists, researchers, healthcare providers and legislature are essential to control the tick's population and reduce the risk of human and animal exposure. Global climate change adds more challenge to tick control efforts are acaridae are spreading into new habitat following their hosts. Also, ticks develop resistance to commonly used acaricides. Therefore, research for novel approaches for acaridae control is the most urgently needed.

The collaborative efforts could include:

1. Funding research for developing and testing novel approaches for tick control (biocontrol, genetic control, etc.).
2. Constant updating the regulatory framework to expedite the approval and implementation of novel effective measures for tick control.
3. Improve collaborative efforts between agencies in charge of tick control.
4. Engage tick controlling agencies in neighboring countries to synchronize their efforts.
5. Engage the custom/trade controlling agencies in controlling the export/import of products having high risk of tick exposure.
6. Advance the efficacy of public education by establishing the state/county legislative initiatives.
7. Engage the local veterinarian and physician in the public education programs. Promote public education through workshops, conferences and flyers.
8. Funding the research to develop vaccines against tick-borne diseases in human and animals.
9. Funding studies to better understand ticks' life cycle to identify novel targets for acaricides.
